# Calcium starvation leads to strain-specific gene regulation of lipid and carotenoid production in *Mucor circinelloides*

**DOI:** 10.1093/g3journal/jkaf207

**Published:** 2025-09-06

**Authors:** Helle Tessand Baalsrud, Dana Byrtusova, Thu-Hien To, Ida Emilie Larsen, Veronica Aarvik Bøe, Lars Grønvold, Juan Fu, Mariann Árnyasi, Volha Shapaval, Simen Rød Sandve

**Affiliations:** Department of Animal and Aquacultural Sciences, Faculty of Biosciences, Norwegian University of Life Sciences, 1432 Ås, Norway; Department of Mechanical Engineering and Technology Management, Faculty of Science and Technology, Norwegian University of Life Sciences, 1432 Ås, Norway; Department of Animal and Aquacultural Sciences, Faculty of Biosciences, Norwegian University of Life Sciences, 1432 Ås, Norway; Department of Animal and Aquacultural Sciences, Faculty of Biosciences, Norwegian University of Life Sciences, 1432 Ås, Norway; Department of Mechanical Engineering and Technology Management, Faculty of Science and Technology, Norwegian University of Life Sciences, 1432 Ås, Norway; Department of Animal and Aquacultural Sciences, Faculty of Biosciences, Norwegian University of Life Sciences, 1432 Ås, Norway; Department of Mechanical Engineering and Technology Management, Faculty of Science and Technology, Norwegian University of Life Sciences, 1432 Ås, Norway; Department of Animal and Aquacultural Sciences, Faculty of Biosciences, Norwegian University of Life Sciences, 1432 Ås, Norway; Department of Animal and Aquacultural Sciences, Faculty of Biosciences, Norwegian University of Life Sciences, 1432 Ås, Norway; Department of Animal and Aquacultural Sciences, Faculty of Biosciences, Norwegian University of Life Sciences, 1432 Ås, Norway; Department of Mechanical Engineering and Technology Management, Faculty of Science and Technology, Norwegian University of Life Sciences, 1432 Ås, Norway; Department of Animal and Aquacultural Sciences, Faculty of Biosciences, Norwegian University of Life Sciences, 1432 Ås, Norway

**Keywords:** comparative genomics, gene regulation, fungi, biotechnology

## Abstract

Fungi are pivotal in transitioning to a bio-based, circular economy due to their ability to transform organic material into valuable products such as organic acids, enzymes, and drugs. *Mucor circinelloides* is a model organism for studying lipogenesis and is particularly promising for its metabolic capabilities in producing oils like TAGs and carotenoids, influenced by environmental factors such as nutrient availability. Notably, strains VI04473 and FRR5020 have been identified for their potential in producing single-cell oils and carotenoids, respectively. Calcium starvation has previously been shown to have strain-specific effects, with VI04473 accumulating more lipids and FRR5020 producing more carotenoids. Here, we used genome sequencing, comparative genomics, transcriptomics, and metabolite phenotyping to explore the genetic basis of lipid and carotenoid production under calcium starvation in these strains. We found extensive genomic rearrangements between these strains, as well as low conservation of gene regulatory responses to calcium depletion. This lack of conservation also applies to genes involved in lipid and carotenoid production, ie the lipidome. Crucially, we identified several metabolic pathways with distinct transcriptional responses to calcium depletion, suggesting the existence of a previously unrecognized, strain-dependent mechanism by which calcium signaling modulates metabolite production. This points to a potentially novel regulatory pathway linking calcium homeostasis to secondary metabolism in fungi, which may be linked to the complex gene family evolution of several lipidome-genes. Our study sheds light on the complexity of the evolution of metabolic networks in *M. circinelloides*. Understanding these genetic underpinnings can optimize the industrial use of *M. circinelloides*, enhancing lipid productivity and stress tolerance, and tailoring metabolic profiles for specific applications.

## Introduction

Fungi play a pivotal part of transitioning from a petroleum-based to a bio-based, circular economy due to their ability to transform organic material to a variety of useful products such as food, beverages, organic acids, enzymes, antibiotics, and drugs (Meyer et al. 2016, 2020; Dzurendova et al. 2022). *Mucor circinelloides* is one such species with immense industrial potential due to its diverse metabolic capabilities to produce high-value oils and other valuable compounds (Shapaval et al. 2014; Carvalho et al. 2015; Dzurendova et al. 2020). The versatility in metabolic pathways stems from *M. circinelloides*’ adaptation to grow in a wide range of environmental conditions and substrates (Vellanki et al. 2018). There are numerous different strains that are ubiquitously found in soil decomposing various organic matter but can also grow on various food products as well as infect plant and animal hosts, including humans (Iturriaga et al. 2012; Soare et al. 2020 ; Dzurendova et al. 2022; Fazili et al. 2022). These strains vary in their ability to produce different compounds under various environmental conditions, for instance, cellular lipids can constitute between 15% (Zhang et al. 2007) and 54% of the total cellular weight (w/w) (Dzurendova et al. 2021a, 2021b), chitin and chitosan biopolymers can account to up to 8% (w/w) (Losada et al. 2025). Although genetic tools such as CRISPR-Cas9 (Nagy et al. 2017, 2019) and genetic transformation (van Heeswijck and Roncero 1984; Torres-Martínez et al. 2012; Nicolás et al. 2018) have already been successfully demonstrated in this species, currently only a few strains have been sequenced, with only one annotated, long-read-based genome assembly (Navarro-Mendoza et al. 2019 ). A better understanding of the genetic basis of this natural variation across multiple strains is needed to harness the biotech possibilities of *M. circinelloides* by genetically engineering strains with enhanced lipid productivity, improved stress tolerance, and tailored metabolic profiles to meet specific industrial objectives.

Two strains have recently been identified as promising industrial candidates: VI04473 for single-cell oil (SCO) production (Shapaval et al. 2014; Carvalho et al. 2015; Dzurendova et al. 2020) and FRR5020 for production of carotenoids (Dzurendova et al. 2021b). SCOs—oils produced intracellularly in lipid bodies—are sustainable alternatives to vegetable, palm, and fish oils for food and feed, and carotenoids are natural pigments with a wide range of applications (Naz et al. 2020). These traits can be influenced by numerous environmental factors such as nutrients availability, pH, and availability of metal ions such as calcium (Ca) (Gorain et al. 2013; Dzurendova et al. 2021b). Nitrogen limitation conditions are known to stimulate lipid accumulation in oleaginous microorganisms where it inhibits cell proliferation and cells redirect resources from protein synthesis to lipid biosynthesis (Carvalho et al. 2015). Recently, Ca starvation was shown to have strain-specific effects on lipid and carotenoid accumulation; in VI04473, Ca starvation led to a higher accumulation of lipids, whereas in FRR5020, there were small changes in lipid content but a large increase in carotenoid content (Dzurendova et al. 2021b). These effects were also partially dependent on phosphorous concentration and, thus, pH, which is known to affect enzyme activity, nutrient availability, and metabolic pathways (Bhalla et al. 2022). Increasing production of lipids and carotenoids under low levels of phosphate is of particular interest to the industry, as phosphate is a limited resource (Drangert 2012). A significant knowledge gap toward this end is understanding the genetic basis of metabolite accumulation at different Ca conditions under low phosphate levels in diverse *M. circinelloides* strains.

In this study, we combined genome sequencing, comparative genomics, transcriptomics, and metabolite phenotyping to investigate the molecular basis for lipid and carotenoid production in two phenotypically divergent strains of *M. circinelloides*: FRR5020 and VI04473. We found large differences in the genome structure of *M. circinelloides* strains as well as potentially causal differences in gene content and gene regulation underlying the extreme strain variability in metabolite production.

## Materials and methods

### Fungal strains and inoculum preparation

The selection of *Mucor circinelloides* strains was based on the results of the previous study performed by Dzurendova et al. (2021). The FRR5020 strain was originally obtained in the Food Fungal Culture Collection (FRR; North Ryde, Australia), and the VI04473 strain was obtained from the Veterinary Institute (VI, Ås Norway). The stock cultures were stored at −80 °C and consisted of asexual fungal spores in a glycerol-NaCl solution. To recover fungal culture from the cryo-vials and prepare spore inoculum, cultivation on malt extract agar (MEA) was done. MEA plates were prepared by dissolving 50 g/L of agar powder (Agar Powder, VWR Chemicals) in deionized water, which was then autoclaved at 115 °C for 15 min and subsequently cooled down to 45 °C before being poured into Petri dishes (VWR, 9 cm diameter). The MEA Petri dishes were stored at 4 °C until use. Before cultivation, the MEA plates were exposed to UV light in a class 2 biological safety cabinet (Cellegard ES) for 15 min to prevent any contamination. Three 5 µL droplets of stock spore suspension (stored in a −80 °C freezer) were inoculated onto each MEA plate. The MEA plates were then incubated at 25 °C for 4 d in a VWR Inco-line 68 R heat locker. The cultivation was performed in polypropylene microtiter plates, which are not transparent to blue light. The cultivation on MEA plates was done in 4 replicates to obtain enough spores for the experiments. For inoculum preparation, 10 mL of sterile saline solution was added to the MEA plates, spores were harvested by mixing and scraping with single-use plastic loops, and then the spore solution was transferred into sterile Falcon tubes. The obtained spore solution was used as inoculum to inoculate media with different Ca and phosphorus concentrations.

### Cultivation under different Ca availability

To investigate the effect of Ca on pigment and lipid production in *Mucor circinelloides* strains FRR5020 and VI04473 under varying phosphorus availability, nitrogen-limited cultivation media were designed using a full factorial approach. The experimental design included 3 concentrations of inorganic phosphorus (Pi), provided as phosphate salts (KH_2_PO_4_ and Na_2_HPO_4_), and 2 Ca conditions: Ca1 (presence) and Ca0 (absence). The nitrogen-limited broth media were used as described by Dzurendova et al. (2021) and contained the following components (g/L): glucose, 80; (NH_4_)_2_SO_4_, 1.5; MgSO_4_·7H_2_O, 1.5; CaCl_2_·2H_2_O, 0.1; FeCl_3_·6H_2_O, 0.008; ZnSO_4_·7H_2_O, 0.001; CoSO_4_·7H_2_O, 0.0001; CuSO_4_·5H_2_O, 0.0001; MnSO_4_·5H_2_O, 0.0001. All chemicals were sourced from Merck (Germany). The reference phosphorus concentration (Pi1) was set at 7 g/L KH_2_PO_4_ and 2 g/L Na_2_HPO_4_, based on its frequent use in the cultivation of oleaginous *Mucoromycota* (Kosa et al. 2018). In addition to Pi1, media were formulated with a higher phosphorus level (Pi4, 4× Pi1) and a lower level (Pi0.5, 0.5× Pi1) to assess the impact of varying Pi availability. Cultivation was carried out using a microtiter plate system (Duetz-MTPS). The microtiter plate system (Duetz-MTPS) consists of deep well 24 square microtiter plates, sandwich covers, and clamp systems to mount microplates on a shaking platform of an incubator. In each well of the microtiter plates, 7 mL of media (Ca1P1, etc.) was inoculated with 5 μL of spore solution, and in total of 3 wells corresponding to biological replicates per treatment were prepared. The microtiter plates were mounted on the shaking platform using clamp systems in a shaker (Kuhnershaker X, Climo-shaker ISF1-X) and incubated at 25 °C and 250 rpm for 5 d. Incubation was done under strictly controlled conditions in incubator shaker without light exposure.

### Biomass preparation for analysis

After the cultivation in Duetz-MTPS, fungal biomass was washed with deionized water using vacuum filtration system. Washed biomass was transferred into sterile Eppendorf tubes (2 mL) and immediately placed on dry ice to prevent biomass degradation. All biomass samples were stored at −80 °C until RNA extraction and chemical analysis. To freeze-dry the biomass for chemical analysis, the tubes with frozen biomass were put into the dryer and maintained at 0.08 bar and −50 °C for 2 d for the water to sublimate. The freeze-dried biomass was kept in the freezer at −20 °C.

### Vibrational spectroscopy analysis

Before Fourier transform infrared (FTIR) spectroscopy analysis, the dried biomass was homogenized: 2 mL screw-cap microcentrifuge tube was filled with 250 mg (710 to 1,180 μm diameter) acid-washed glass beads (Sigma-Aldrich, USA) and a small amount of biomass was then added to the tube, followed by 0.5 mL of distilled water. The tubes were placed in a Precellys Evolution tissue homogenizer (Berlin Instruments, France) at 5,500 rpm, 20 s cycle length, and 6 cycles (2 × 20 s, 3 runs) to ensure complete homogenization of the biomass. Approximately 10 μL of homogenized biomass was pipetted onto an IR transparent 384-well silica microplate, with 1 free spot between each sample. Three technical replicates were pipetted onto 384-well FTIR silica microplate, for each sample. After drying at room temperature for 30 to 45 min, the samples were measured in FTIR spectrometer. FTIR spectra were recorded in transmission mode using a high-throughput screening extension (HTS-XT) unit coupled to a Vertex 70 FTIR spectrometer (both Bruker Optik GmbH, Leipzig, Germany). Spectra were recorded in the region between 4,000 and 500 cm^−1^, with a spectral resolution of 6 cm^−1^, a digital spacing of 1.928 cm^−1^, and an aperture of 5 mm. For each spectrum, 64 scans were averaged. Spectra were recorded as the ratio of the sample spectrum to the spectrum of the empty IR transparent microplate. In total, 63 FTIR biomass spectra were obtained. The OPUS software (Bruker Optik GmbH, Leipzig, Germany) was used for acquisition and instrument control.

FTIR spectra were analyzed using Orange 3.16 (Demšar et al. 2013). For the estimation of lipid-to-protein ratios, FTIR spectra were preprocessed using extended multiplicative signal correction (EMSC) with linear, quadratic, and cubic terms (Forfang et al. 2017). The calculations of lipid-to-protein ratios were done in Microsoft Excel (Microsoft Excel 2019, Microsoft Corp., Redmond, WA, USA) by dividing the relative absorbance values for the –C=O stretching peak at 1,745 cm^−1^ (correlate with the total lipid content in cells) by the Amide 1 peak at 1,650 cm^−1^ (reference band for biomass).

To determine the total relative carotenoid content in the biomass, analysis using a FT-Raman spectroscopy was performed. The freeze-dried biomass was transferred into 2 mL screw vials with 400 μL glass inserts until the bottom of the vials was completely covered with biomass, making 3 technical replicates for each sample. The samples in glass vials were put into a 96-well sample box, for 400 μL flat bottom glass insert and then placed onto a V-grooved sample mount with the samples facing the lens assembly in the MultiRAM FT-Raman spectrometer (Optic GmbH, Germany). The first and the last sample vials contained acetonitrile, used for reference measurements.

FT-Raman spectra were analyzed using Orange 3.16 (University of Ljubljana, Slovenia) (Demšar et al. 2013, Toplak et al., 2017). For the estimation of the relative carotenoids content, FT-Raman spectra were preprocessed using EMSC with linear, quadratic, and cubic terms (Dzurendová et al. 2021). The calculations were done in Microsoft Excel (Microsoft Excel 2019, Microsoft Corp., Redmond, WA, USA) by dividing the Raman intensities at 1,523 cm^−1^ (related to carotenoids) and 1,445 cm^−1^ (related to biomass).

### Lipid extraction and fatty acid profile analysis

Analysis of the total lipid content and fatty acid profile was performed according to methodology in (Langseter et al. 2021). About 20 mg of the freeze-dried biomass together with approximately 250 mg (710 to 1,180 um diameter) of acid-washed glass beads (Sigma-Aldrich, USA) were filled in a 2 mL screw-cap polypropylene tube. To the mix, 500 μL chloroform was added, and 100 μL of the internal standard was pipetted with a Hamilton syringe. The mix was then homogenized in a Precellys Evolution tissue homogenizer (Berlin Instruments, France) at 5,500 rpm, 20 s cycle length, and 6 cycles (2 × 20 s, 3 runs). The biomass was transferred into glass reaction tubes by washing the polypropylene tube 3 times with 800 μL methanol-chloroform-hydrochloric acid solvent mixture (7.6:1:1 v/v). After washing the glass reaction tubes, 500 μL of methanol was added into them. The mixture was incubated at 90 °C for 90 min in a Stuart SBH130D/3 block heater (Cole-Parmer, UK). After the samples were cooled down to room temperature, a small amount of sodium sulfate was added together with 1 mL of distilled water. The mixture was cooled down to room temperature after incubation. To extract the fatty acid methyl esters (FAMEs), 2 mL of hexane was added, and vortex mixed for 10 s before centrifugation at 3,000 rpm for 5 min at 4 °C. The upper organic phase was collected in glass tubes. The upper phase was extracted 2 more times, and a 2 mL hexane-chloroform mixture (4:1 v/v) was added. To evaporate the solvent, the glass tubes were placed in a Stuart SBH130D/3 block heater (Cole-Parmer, UK) connected to nitrogen at 30 °C. The FAMEs were transferred into gas chromatography (GC) vials by washing the glass tubes 2 times with 750 μL hexane (containing 0.01% butylated hydroxytoluene [Sigma-Aldrich, USA]), followed by pipette mixing approximately 15 times. The GC vials were stored in a −20 °C freezer until GC-flame ionization detector (FID) analysis.

Determination of total lipid content and fatty acid composition was performed by using gas chromatography 7820A System (Agilent Technologies, USA), equipped with an Agilent J&W 121–2323DB-23 column, 20 m × 180 μm × 0.20 μm and an FID. Helium was used as a carrier gas. The total run time for 1 sample was 36 min with the following oven temperature increase: initial temperature 70 °C for 2 min, after 8 min to 150 °C with no hold time, 230 °C in 16 min with 5 min hold time, and 245 °C in 1 min with 4 min hold time. The injector temperature was 250 °C and 1 μL of a sample was injected (30:1 split ratio, with split flow 30 mL/min). For the identification and quantification of fatty acids, the Supelco 37 Component FAME Mix (C4–C24 FAME mixture, Sigma-Aldrich, USA) was used as an external standard, in addition to C13:0 TAG internal standard. Measurements were controlled by the AgilentOpenLAB software (Agilent Technologies, USA).

### DNA isolation and whole-genome sequencing

DNA isolation started from flash-frozen fungal tissue. The sample was homogenized by grinding under liquid nitrogen. DNA was isolated using the Nucleobond HMW DNA extraction kit (Macherey-Nagel) following the manufacture protocol with small modification. For lysis, sample was incubated at 50 °C for 2.5 h, and all centrifugation steps were done at minimum 10,000 g. Quality of the DNA was evaluated by running the sample on 0.5% agarose gel and by using a Nanodrop 8000 Spectrophotometer. After DNA isolation, size selection was performed by using an short read eliminator kit (PacBio). Oxford Nanopore technology (ONT) library preparation was done using the Native Barcoding Kit 24 V24 (SQK-NBD114.24) (Oxford Nanopore) following the manufacture protocol. The library was loaded on an R9.4.1 flow cell and was sequenced using a Promethion24 device. To maximize the data output, we performed a nuclease flush when the ratio of the sequencing pores dropped under 20%. Nuclease flush and library reloading were repeated twice. Guppy 5.1.13 was used for base calling with the high-accuracy base calling model.

### Genome assembly and annotation

The genome assembly was done by first by removing short reads (<4,000 bp) and low-quality ones (q < 7), then assembled with Flye version 2.9.1 (Kolmogorov et al. 2019) and Canu version 2.2 (Koren et al. 2017). The default correctedErrorRate for Nanopore reads is 0.144, which might be too conservative for the newer chemistries of ONT. We therefore tested several values for this parameter (default = 0.144, 0.1, 0.05, and 0.03). The assemblies were then evaluated with Inspector version v1.0.2 (Chen et al. 2021 ) and Merqury version v1.3 (Rhie et al. 2020) for accessing the quality (QV) and completeness; and with BUSCO version 5.4.3 (Manni et al. 2021) for accessing the quantity of conserved genes. Based on assessing correctness, contiguity, and completeness for all assemblies, we selected the Canu assemblies with correctedErrorRate = 0.03.

Annotation was done with the funannotate pipeline version v.1.8.13, (Jonathan and Jason 2020) using the RNA-seq data generated in this project. This pipeline was developed to annotate fungi and is widely used. RNA-seq samples were merged to the corresponding strain to serve as evidence for the annotation. The assemblies were first cleaned to remove the repetitive contigs, and then masked the repeat regions using the default masker tantan in funannotate. The gene models were predicted with several methods and then combined into one by Evidence Modeler. Functional annotation was run with all the implemented tools in the pipeline: Pfam, InterProScan, Eggnog, UniProtKb, Pobius, antiSMASH, and MEROPS. Centromeres were manually annotated using BLAST+ v.2.14.1 (Altschul et al. 1990) with sequences of known centromere motifs and centromere-specific transposable elements (GREM-LINEs) from (Navarro-Mendoza et al. 2019). Telomeres were annotated by searching for the motif TTAGGG (Nierman et al. 2005).

### Comparative genomics

We plotted synteny between *M. circinelloides* strains for which we had chromosome-level assemblies: FRR5020, VI04473, and MU402 using GENESPACE v.1.4 (Lovell et al. 2022). A synteny riparian plot was generated using VI04473 as a reference. MU402 was used as an outgroup in these analyses to investigate genomic rearrangements between FRR5020 and VI04473.

As the analyses above only included 3 genomes, we carried out a more thorough phylogenomic investigation also including species that do not have chromosome-level assemblies, but do have gene annotations. We downloaded protein sequence data based on genome annotations from the Ensembl collection number 54 for *Mucoromycota* (http://ftp.ensemblgenomes.org/pub/fungi/release-54/fasta/fungi_mucoromycota1_collection/). This included an additional strain of *M. circinelloides* which we denoted MucCirc4. We also downloaded the protein sequences from *M. circinelloides f. lusitanicus* CBS277.49 (Navarro-Mendoza et al. 2019), hereafter denoted MU402. We excluded *Mortierella verticillata* (GCA000739165) because it contained very few genes (1,523). As outgroup species we included *Conidiobolus coronatus*, *Aspergillus nidulans*, and *Saccharomyces cerevisiae.* Including FRR5020 and VI04473, this resulted in a dataset of a total of 30 species (see Supplementary Table 4 for assembly versions and data location). Based on this dataset, we used Orthofinder2 (Emms and Kelly 2015, 2019) to investigate the phylogenetic placement of our 2 *Mucor circinelloides* strains (combining phylogenetic signals from conserved ortholog gene protein sequences) as well as gene family size evolution. Please see the extensive documentation of Orthofinder2 for details.

### RNA isolation and transcriptomic sequencing

RNA was isolated for both strains and both experiments (6 + 2 treatments, 4 replicates for each). The RNA extraction was performed with the RNeasy Plus Mini kit. Concentration and integrity of RNA were determined using the 4150 TapeStation system and Nanodrop 8000 (Thermo Fisher Scientific). Most samples had RNA integrity number values higher than 6. Preparation of sequencing libraries and RNA sequencing was carried out at Novogene. Illumina sequencing libraries (150 PE) were prepared with the TruSeq Stranded mRNA Library kit according to the standard protocol. Libraries were sequenced using the Illumina HiSeq 2500 platform.

### Differential gene expression analyses

We obtained sufficient RNA-seq data for at least 3 replicates from each treatment from each strain from each experiment (Supplementary Fig. 4 and 5). RNA sequencing data were processed for each strain separately using the nf-core/rna-seq v3.11.2 pipeline (Ewels et al. 2020). This pipeline includes the following steps: raw reads underwent quality and adapter trimming with Trim Galore, removal of genome contaminants with BBSplit, removal of ribosomal RNA with SortMeRNA, alignment to their respective genome assembly using STAR, and quantification with Salmon. Alignments were sorted and indexed with SAMtools, unique molecular identifiers-based deduplication was carried out with UMU-tools, and duplicates were marked with Picard MarkDuplicates. StringTie was used for transcript assembly and quantification and bigwig coverage files were generated with BEDTools and bedGraphToBigWig. Extensive quality control was carried out for each sample and collated with MultiQC. Differential expression analyses were carried out using DESeq2 package (Love et al. 2014) in R. Ca0 vs Ca1 and the P1 vs P4 contrasts were investigated to represent more stressful vs less stressful conditions, respectively. We compared gene expression between strains using 1:1 orthologs defined by Orthofinder. We categorized differentially expressed genes (DEGs) based on how conserved gene regulation was between strains, ie whether they were significant in only 1 strain, significant but opposite directions in strains, or significantly up or down in both strains.

### Candidate gene analysis

We used lipidome-genes annotated for the strain MU402 in Sokołowska et al. (2023). To annotate lipidome-genes in FRR5020 and VI04473, we identified orthogroups that contained MU402 lipidome-genes. FRR5020 and VI04473 genes within those orthogroups were assigned as lipidome orthologs of the closest MU402 lipidome-gene based on the orthogroup tree distance. We manually inspected candidate genes known to be related to Ca levels, lipids, and carotenoid pathways (Supplementary Table 6). Some of the genes were not properly annotated in both strains—these were manually annotated using BLAST.

### Gene regulation synteny analysis

For each 1:1 ortholog, we compared the syntenic bulk identity from GENESPACE (see above) between FRR5020 and VI04473. We then interlaced this with the differential expression analyses Ca0 vs Ca1 and the P1 vs P4 contrasts. We classified 1:1 orthologs between FRR5020 and VI04473 based on how many orthologous neighbors they share (0, 1, or 2 neighbors). For each category (0, 1, or 2 neighbors), we did a linear regression of log_2_ fold changes (LFC) between strains FRR5020 and VI04473. We also investigated if the different categories of conservation of DEGs (see above) were in smaller synteny bulks or closer to a synteny bulk breakpoint. For all 1:1 orthologues in genomic regions <10,000 bp from a synteny breakpoint, we investigated the difference in LFC between strains as a function of distance to the closest.

## Results

### Phenotypic divergence between strains in response to Ca starvation

**Fig. 1.**
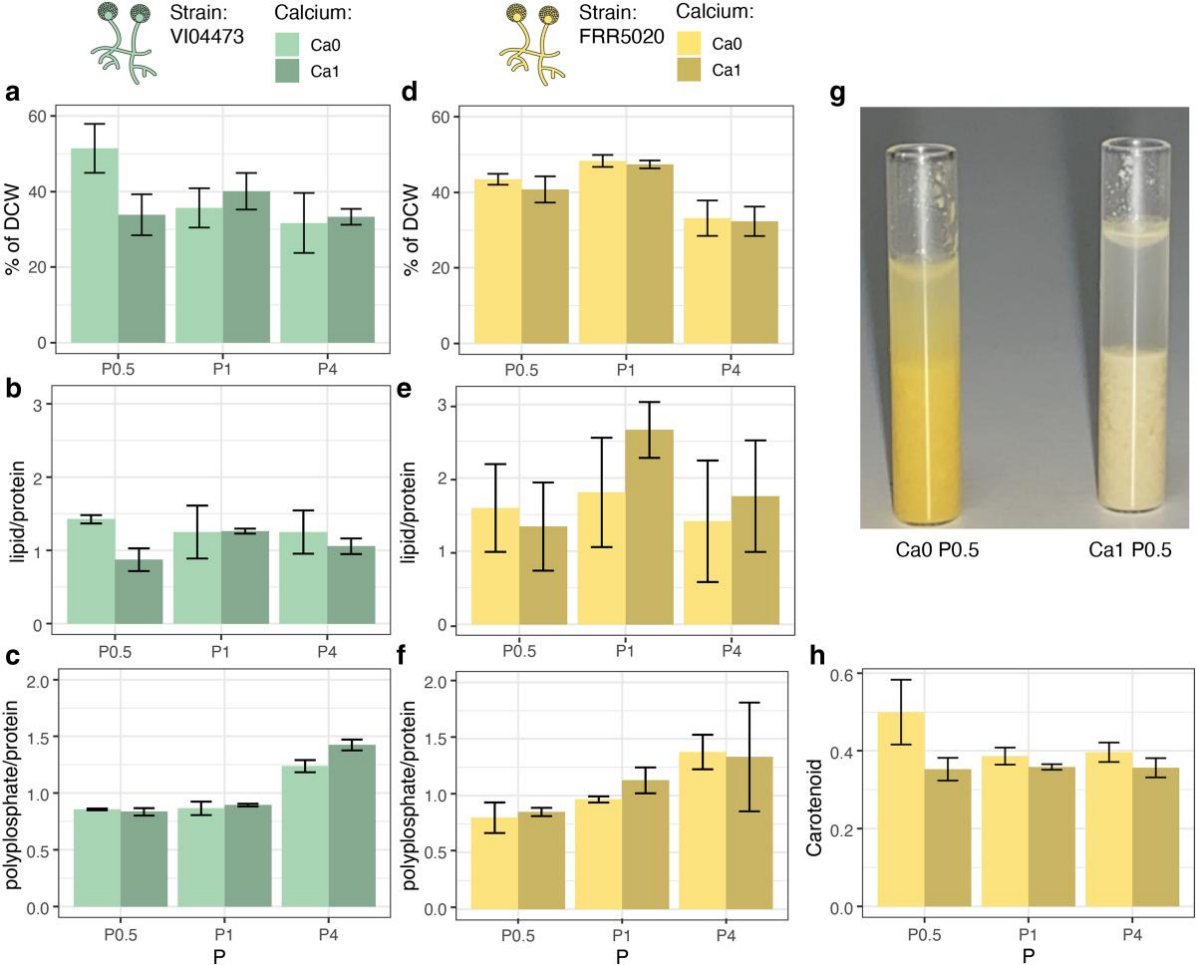
The effect of Ca and P on lipids, proteins, polyphosphates, and carotenoids in *Mucor circinelloides*. Strain (FRR5020 and VI04473) and Ca (Ca) level is colored according to the legend, with Ca1 being the reference level of Ca and Ca0 is absence of Ca in the media. Phosphate (P) has 3 levels, P1 is the reference level and P0.5 and P4 is half or 4 times that level, respectively. a) and d) show lipid content as a percentage of DCW, b) and e) are the lipids-to-protein ratio, c) and f) are the polyphosphates-to-proteins ratio, g) is a picture of tubes containing tissue of FRR5020 with or without Ca at P0.5 level, and h) is the level of carotenoids in the FRR5020 strain quantified by the RI_1523_/RI_1445_ ratio. For pictures of all replicates, see Supplementary Fig. 2. Raw data are available in Supplementary Tables 1 to 3.

**Fig. 2.**
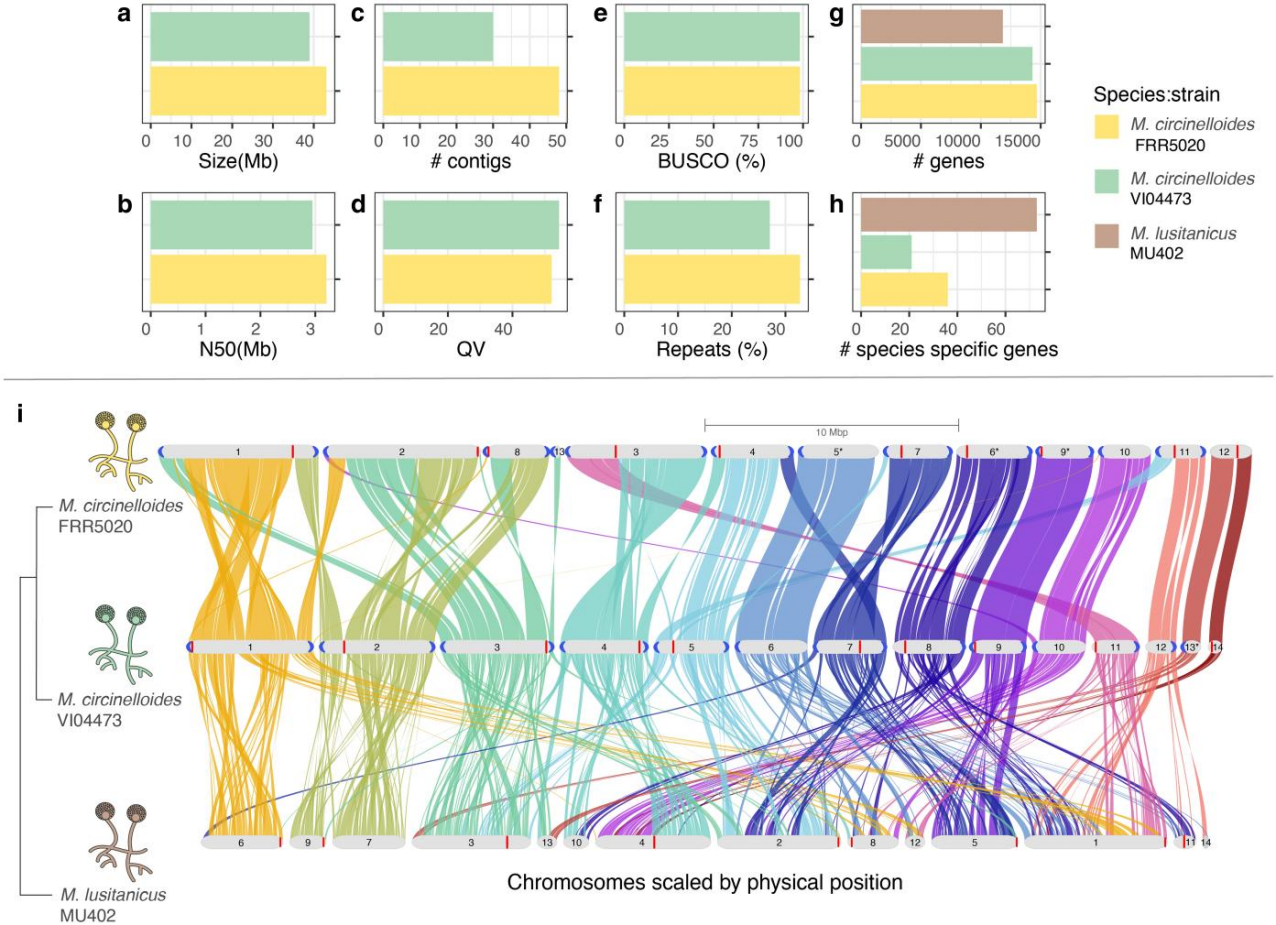
Comparison of chromosome-level *Mucor* genome assemblies. Barplots showing assembly statistics for the two *M. circinelloides* strains, FRR5020 and VI04473, including a) genome size (Mb), b) contig N50 (MB), c) total number of contigs, d) assembly quality value (QV), e) BUSCO completeness score and f) repeat content. Comparative genomic statistics for FRR5020 and VI04473, and *Mucorlusitanicus* strain MU402 with g) the total number of genes for each strain, h) the number of species-specific genes for each strain, and i) Genome-wide synteny relationships between the three strains. The plot was generated by GENESPACE (Lovell et al. 2022). The phylogenetic tree was generated by Orthofinder (see Supplementary Fig. 3 for the full tree). Chromosomes are ordered horizontally to maximize collinearity with the genome assembly for the VI04473 strain. Only chromosomes or scaffolds with >100 genes and collinearity blocks > 5 genes were included in the plot. The size of the putative chromosomes are scaled by Mbs according to legend. Braids illustrate gene order along the chromosome sequence. *Indicate inverted chromosomes. Centromeres are indicated as vertical red bars, telomeres are highlighted at chromosome ends in blue.

To characterize lipid and carotenoid production in VI04473 and FRR5020, we applied the same methods as previously described by Dzurendova et al. (2021). The average lipid content ranged from 24% to 59% of the dry cell weight (DCW), with the highest lipid content found in VI04473 (Fig. 2a and d). As expected, we observed marked differences in lipid accumulation ability between strains. VI04473 displayed Ca-dependent changes in lipid accumulation with negligible effects of different P levels. At a Ca starvation (Ca0) and P limitation (P0.5), VI04473 displayed an increase in lipid content from 34% to 51% of DCW, while at other P levels, the increase in lipid content was not observed as it was reported by Dzurendova et al. (2021). Lipid accumulation in FRR5020, on the other hand, is not influenced by Ca availability but decreases at high levels of P (Fig. 2d). Biomass production was in accordance with the previously published results (Dzurendova et al. 2021b), where decrease in P resulted in the decrease in biomass for both strains when Ca was present. The same was observed FRR5020 under Ca deficiency, while it was relatively stable for VI04473. The lipids-to-proteins ratio was also affected positively by Ca starvation at P0.5 in VI04473, with no effect at P1 and P4 (Fig. 1b). In FRR5020, there was a large variability in lipids-to-proteins ratio, with no apparent effect of Ca (Fig. 1e). The level of Ca did not have any discernible effect on the fatty acid profiles (Supplementary Fig. 1). In summary, our experiments reproduced previous findings (Dzurendova et al. 2021b) of strain-specific differences in Ca starvation–dependent lipid accumulation, but only at low levels of P.

For the high pigment producing strain, FRR5020, there was a significant effect of Ca starvation on pigmentation levels (Fig. 1g and h). Carotenoid content in Ca0P0.5 was significantly higher than for all other treatments, with a mean value of RI_1523/RI_1445 of 0.5 (Fig. 1g). This effect of Ca at the P0.5 level is highly visible, with biomass grown at Ca0 being a bright yellow color as opposed to milky yellow at Ca1 (Fig. 1h and Supplementary Fig. 2).

### Chromosome-level assemblies for two *M. circinelloides* strains

To investigate genetic differences between strains we generated highly contiguous genome assemblies meeting the quality standards of the Earth Biogenome Project for both VI04473 and FRR5020, with contig N50 ≥3 Mb, QV ≥47, and BUSCO ≥98 (Fig. 2 and Supplementary Fig. 3). Canu generated better assemblies than Flye, possibly because of the step in Canu that performs error correction of reads. Total length of the 2 assemblies were very similar, 43 and 39 Mb for FRR5020 and VI04473, respectively, which is slightly larger than the high-quality genome of *M. lusitanicus* strain MU402 (37 Mb) (Navarro-Mendoza et al. 2019). The difference in assembly length between strains is largely due to a difference in repeat content between the 2 assemblies, with FRR5020 harboring 14.10 Mb repeats (32.71%) and VI5020 harboring 10.55 Mb repeats (27.08%) (Fig. 2f). We investigated the different repeat classes identified by RepeatMasker, and FRR5020 had more repeats that are not assigned to a specific class, ie unknown, as well as having more rRNA repeats (Supplementary Fig. 8). The Canu assemblies were annotated using RNA-seq data using the funannotate pipeline, resulting in 14,677 genes in FRR5020 and 14,313 genes in VI04473. This included rRNA genes for both assemblies. Karyotype is unknown in this species, but the highly contiguous assemblies contained 13 (FRR5020) and 14 (VI04473) gene-containing contigs. We were able to annotate telomeres for most of these contigs (Fig. 2), making our assemblies close to telomere-to-telomere. We also annotated centromeres based on homology with centromere sequence motifs from MU402 (Navarro-Mendoza et al. 2019) (Fig. 2i) and found a mixture of meta-, acro-, and telocentric centromere positions.

### Extensive genomic rearrangements between *M. circinelloides* strains

We used Orthofinder (Emms and Kelly 2019) to determine the phylogenetic relationship between the VI04473 and FRR5020 strains and compare gene family content using all protein-coding genes across all the publicly available annotated *Mucoromycota* genomes as well as outgroups (30 species/strains in total, see Supplementary Fig. 3 and Table 4). Phylogenomic analyses showed that FRR5020 is the sister to *M. circinelloides* 1006phl, and both form a monophyletic clade with VI04473 (Supplementary Fig. 2). *M. lusitanicus* and *Mucor ambiguus* form the sister group to *M. circinelloides*.

The gene content is highly similar between VI04473 and FRR5020. Given the collection of 30 fungi genomes available, we identified 13 species-specific orthogroups containing 36 species-specific genes in the FRR5020 genome, and 10 species-specific orthogroups containing 21 species-specific genes in the VI04473 genome (Fig. 2 and Supplementary Fig. 3).

We also investigated genome-wide synteny between the 2 *M. circinelloides* strains and 1 *M. lusitanicus* strain with high-quality genome assemblies (FRR5020, VI04473 and MU402) (Fig. 2i). A large amount of rearrangement could be seen between MU402 and our *M. circinelloides* assemblies while the synteny between FRR5020 and VI04473 was much more conserved. Major synteny breakpoints between FRR5020 and VI04473 were found at centromere positions in 4 out of the 12 FRR5020 chromosome-level scaffolds, suggesting that centromeres could be hotspots for chromosome reorganizations.

### Low conservation of gene expression response to Ca starvation between strains

We carried out differential expression analyses for each strain separately to identify which genes are differently expressed in response to Ca starvation. As the main phenotypic differences in lipid and carotenoid accumulation between strains are between growth media with Ca0P0.5 and Ca1P0.5 (Figure 1), we focused on this contrast to investigate the effect of Ca starvation on gene expression. This also allowed us to combine expression data from 2 independent experiments (Supplementary Fig. 4 and 5). For each strain, we identified DEGs between different Ca levels using DESeq2 (Love et al. 2014). For VI04473, out of 11,653 expressed genes, 1,524 DEGs were upregulated and 1,743 DEGs were downregulated in response to Ca starvation (*P*_adj_ < 0.1) (Supplementary Fig. 6). For FRR5020, out of 11,765 expressed genes, 1,752 DEGs were upregulated and 2,016 DEGs were downregulated (*P*_adj_ < 0.1) (Supplementary Fig. 6).

**Fig. 3.**
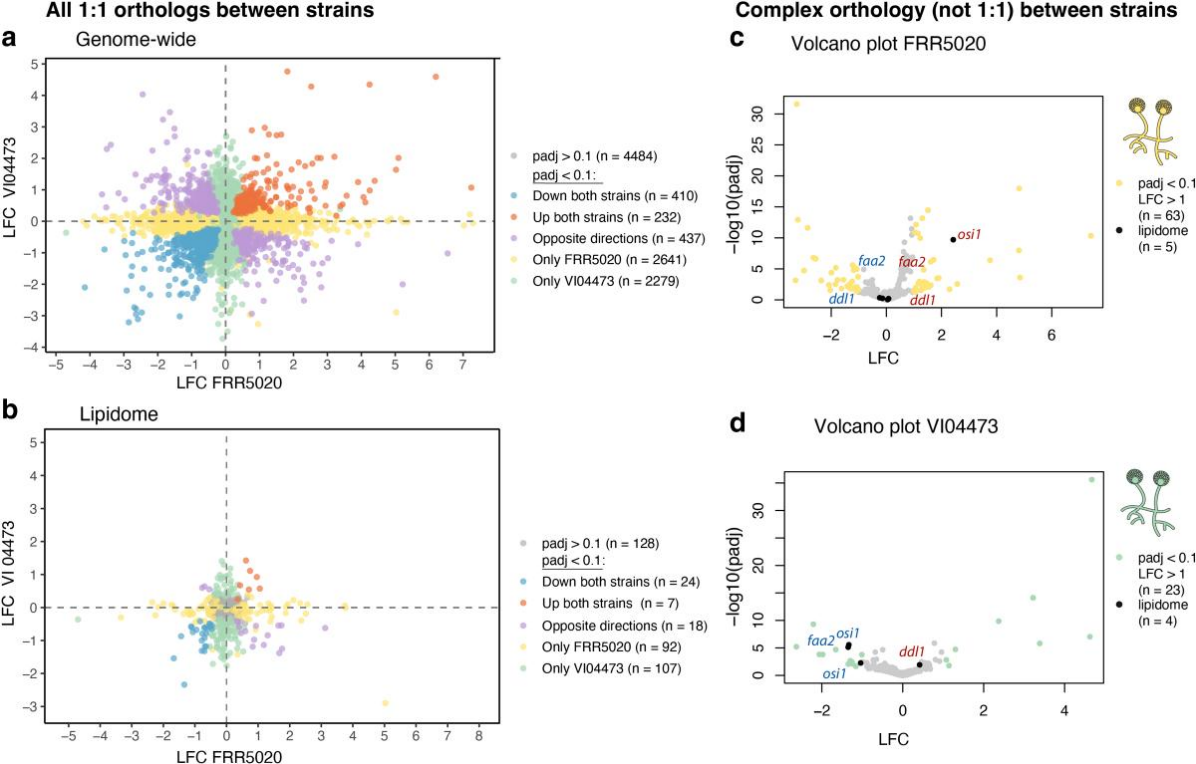
Gene expression results. Correlation of LFC between VI04473 and FRR5020 for all 1:1 orthologs in a) and for lipidome-gene orthologs in b). Genes are grouped according to legend. LFC for genes with more complex, not 1:1 orthology relationships, is shown as volcano plots for c) FRR5020 and d) VI04473. Lipidome-genes are highlighted in black.

To identify gene regulatory differences that could explain strain differences in the phenotypic response to Ca starvation, we first compared LFC in gene expression for all 1:1 orthologous genes (ie genes with single copies in both strains). There were 104,831:1 orthologs in our expression dataset (for an overall overview of ortholog relationships, see Supplementary Table 5). Although we found a weak significant correlation (slope = 0.047, *P* = 2.382 × 10^−11^) between Ca responses in the 2 strains, the R^2^ was extremely low (R^2^ = 0.004) (Fig. 3), and the vast majority of the statistically significant DEGs (*P*_adj_ < 0.1) were only significant in 1 strain (4,920 out of 5,999). Of the DEGs, 437 were significant in both strains but displayed opposite direction of LFC. Only 642 significant DEGs showed the same direction of expression change in the 2 strains, with 232 downregulated and 437 upregulated DEGs under Ca starvation (Fig. 3). These results show that the genome-wide gene regulatory response to Ca starvation was highly strain-specific with only a minority of genes with conserved direction of gene expression. The lack of conservation could be because Ca starvation is particularly stressful for the cells triggering a chaotic gene expression response. To test this hypothesis, we also checked the between-strain correlation of gene expression between the P1 and P4 treatments, which both represent less stressful conditions for fungi. This revealed a much higher similarity (R^2^ = 0.63) between strain gene expression (Supplementary Fig. 7), supporting our stress hypothesis.

Furthermore, we wanted to investigate the regulatory differences between strains for genes specifically related to lipid and carotenoid synthesis. We annotated genes involved in lipid metabolism, ie the “lipidome” in FRR5020 and VI04473 based on orthology to 389 known lipidome-genes in the strain MU402 (Sokołowska et al. 2023) (see the Materials and Methods section). The lipidome also includes genes related to carotenoid synthesis, and these processes are therefore inextricably linked (Sokołowska et al. 2023). In concurrence with the genome-wide results, there was no correlation of LFC in the 371 lipidome-gene 1:1 orthologs between strains (Fig. 3b). Only 31 DEGs showed a similar direction of regulation, with 24 upregulated DEGs and 7 downregulated DEGs in both strains due to Ca starvation.

Due to strain-specific gene family expansion and contraction events, some genes were not in the set of 1:1 orthologs. We therefore investigated in more detail the genes with more complex gene orthology relationships (ie not 1:1 orthologs between the 2 strains). Out of 601 such genes in FRR5020 and 10 such genes in VI04473, 493 and 371 were included in out expression dataset, respectively. For these complex orthology genes, there were 63 DEGs in FRR5020 (Fig. 3c) and 23 DEGs in VI04473 (Fig. 3d) with a LFC higher than 1 (*P*_adj_ < 0.1). Three lipidome-gene orthogroups had significant DEGs in both strains, displaying different regulation of gene duplicates within and/or between strains: *faa2*, *ddl1*, and *osi1* (Fig. 3c and d). The orthogroup with the *faa2* (medium-chain fatty acid CoA ligase) gene family included FRR5020 gene duplicates regulated in opposite directions and 1 downregulated VI04473 gene copy in response to Ca starvation. This could lead to less degradation of medium-chain fatty acids. The second lipidome-gene orthogroup included the yeast ortholog *ddl1* (phospholipase), where the duplicates were also regulated in opposing directions in FRR5020, and a single gene in VI04473 was upregulated. *ddl1* leads to the enhanced function of a vital protein synthesis protein encoded by *ef2* (elongation factor 2), which was downregulated in VI04473. The third orthogroup included the yeast ortholog *osi1* (oxidative stress induced 1), which is related to stress responses in yeast (Gruhlke et al. 2017). Both duplicates were downregulated in VI04473, whereas the single copy in FRR5020 was upregulated.

### Divergent regulation of lipid and carotenoids pathways under Ca starvation

**Fig. 4.**
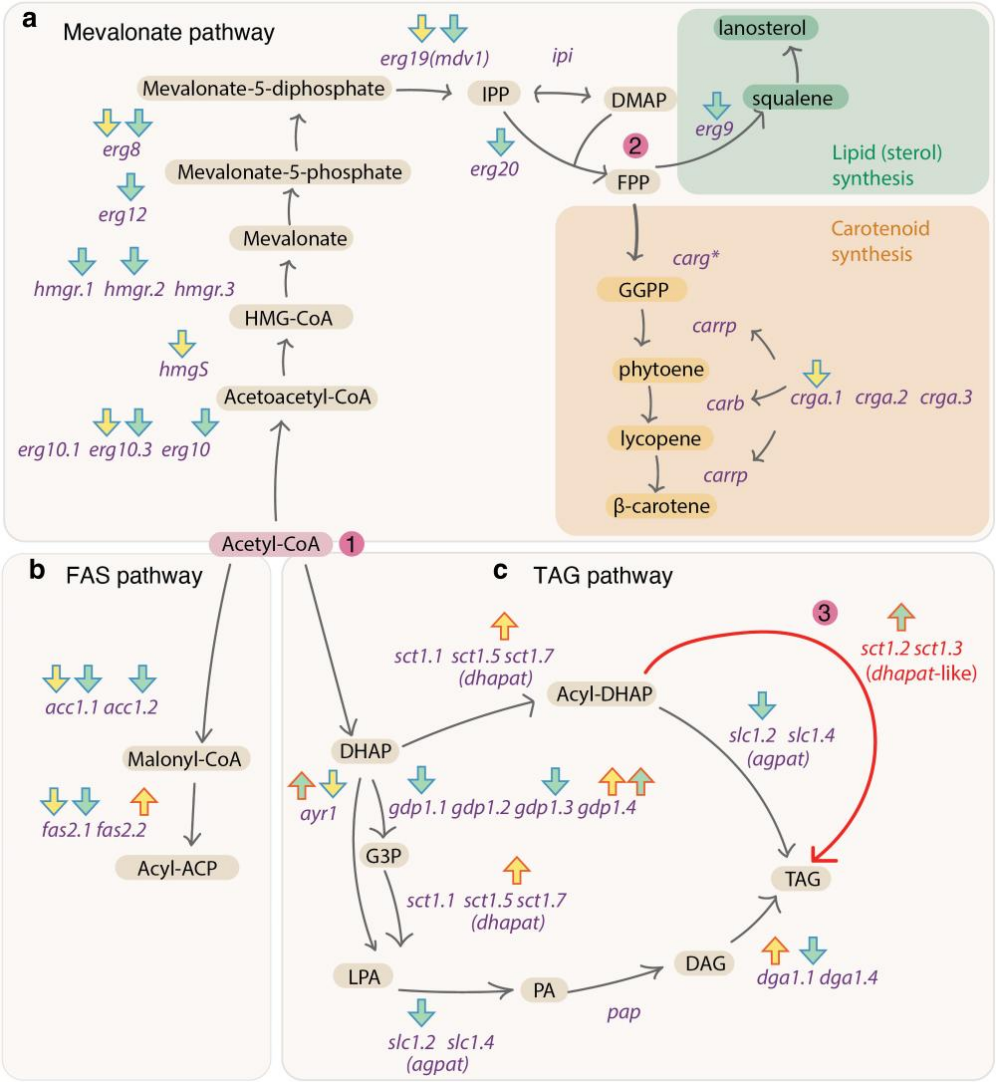
Key lipid pathway regulation under Ca starvation. a) Mevalonate pathway, b) FAS pathway, and c) TAG pathway. Compounds are written in black and genes in purple. Arrows indicate if the gene is significantly downregulated (blue outline) or upregulated (red outline); the fill of the arrow indicates strain, with FRR5020 in yellow and VI04473 in green. The numbers highlighted in pink illustrate key points: 1) Acetyl-CoA can be utilized either in the mevalonate, FAS, or TAG pathways. 2) FPP can be utilized to produce sterols or carotenoids. 3) indicates our proposed alternative pathway to TAG formation with the direct conversion of Acyl-DHAP via *dhapat*-like enzyme.

There is a trade-off in the production of some lipids (eg sterols, TAGs, fatty acids, and phospholipids) and carotenoids as they derive from acetyl-CoA through the TAG, FAS, and mevalonate pathways (Ranganathan et al. 2020). Therefore, we manually investigated the regulatory differences of these pathways under Ca starvation in the different strains (for a complete overview of candidate genes, see Supplementary Table 6). The mevalonate pathway leads to the synthesis of either carotenoids or lipids in the form of sterols (Avalos and Carmen Limón 2015; Sokołowska et al. 2023). Many mevalonate genes were differentially expressed in one or both strains because of Ca starvation (Fig. 4a). In VI04473, the entire pathway is downregulated. There were 3 copies of *hmgr*, in which 2 were downregulated in VI04473. *hmgr* is a known rate-limiting step in the mevalonate pathway (Basson et al. 1986). In FRR5020, some of the genes upstream of *fpp* were downregulated, but not *hmgr.* The pathway junction following the *fpp* step had a clear divergent gene regulatory pattern between the 2 strains. In VI04473, the *erg9* gene was downregulated, which could have led to less squalene, which is a precursor to various forms of sterols (Sokołowska et al. 2023). In FRR5020, 1 of the 2 *carg* paralogs was downregulated leading to the carotenoid synthesis pathway, as well as 1 of the 3 *crga* paralogs. *crga* is a negative regulator of carotenogenesis by blocking transcription of *carb* and *carrp*. Another negative regulator, which is light activated, is white collar protein genes (*wc*) (Silva et al. 2006). There were 4 copies of *wc* in both strains, one of which was upregulated in VI04473. In FRR5020 1 *wc* copy was upregulated and 2 downregulated, and the overall expression level indicated downregulation of this protein. The *dgk1* gene, which plays a role in mevalonate regulation in yeasts (Ranganathan et al. 2020), was not affected by Ca in either strain.

The mevalonate pathway begins with 3 molecules of Acetyl-CoA, which condense to form HMG-CoA, and eventually mevalonate via HMG-CoA reductase. The downregulation of the mevalonate pathway in VI04473 likely freed a lot of Acetyl-CoA for other lipid pathways. The FAS pathway was downregulated in VI04473, and mostly downregulated in FRR5020, except for one of the *fas2* paralogs (Fig. 4b). This could have directly affected synthesis of TAGs and led to the accumulation of MAGs and DAGs (Fig. 4b). Acetyl-CoA can also be used in the TAG pathway directly (Fig. 4c). This pathway is a bit more complex to interpret. For VI04473, the downregulation of 2 of the *gdp1* paralogs and *agpat* would have led to less DAG and TAG. Interestingly, we detected 5 genes annotated as *dhapat* in both strains, with 3 being homologous to *dhapat* in *S. cerevisiae* (*sct1.1*, *sct1.5*, and *sct1.7*). However, 2 copies (*sct1.2* and *sct1.3*) were in orthogroups with only *Mucoromycota* species and no *S. cerevisiae* ortholog—we call these *dhapat*-like. These *Mucoromycota*-specific *dhapat* genes all contained a phospholipid/glycerol acyltransferase domain. In mammalian adipocytes, *dhapat* can lead to direct synthesis of TAG from Acyl-DHAP. We have indicated this route as a possible hypothesis for lipid accumulation in *M. circinelloides*. In the case of VI04473, this would be a primary route for increase lipid accumulation under Ca deficiency.

### Regulatory differences between Ca-sensitive candidate genes

Ca ions are universal second messengers in eukaryotic cells (Roy et al. 2020). When intracellular Ca levels spike (often in response to stress or nutrient signals), they activate Ca-binding proteins (like calmodulin), which then regulate downstream Ca-sensitive genes. Ca-sensitive genes regulate lipid synthesis by (i) controlling enzymes via Ca-dependent signaling, (ii) modulating transcription of lipid genes, (iii) reacting to endoplasmic reticulum and membrane stress, and (iv) influencing lipid-derived secondary messengers. Ca clearly had divergent phenotypic and gene regulatory effects in the 2 *M. circinelloides* strains. As given the wide-ranging effects of Ca on gene regulation (Roy et al. 2020), it can be challenging to disentangle the cause of this divergence. We, therefore, manually inspected candidate genes (Supplementary Table 6) to investigate genes known to be related to Ca levels. Several genes in the calcineurin signaling pathway were differentially regulated in both strains. A gene involved in Ca influx in response to environmental stresses, *cch1* (Calcium Channel Homolog) (Locke et al. 2000), was upregulated in VI04473 and downregulated in FRR5020. Downstream of this gene is *cmk21* (calmodulin-dependent protein kinase), which had 4 copies in both strains, 2 of which were upregulated. One of the 2 calcineurin subunits, *cnb*, was downregulated in FRR5020. The alpha subunit has 3 paralogs in both strains, denoted *cna_a*, *cna_b*, and *cna_c*. *cna_b* was upregulated in VI04473. The expression of 2 genes—*pka* (protein kinase A) and *byca* (gene encoding an amino acid permease)—in the opposite direction from calcineurin genes is associated with regulation of dimorphic switching (Valle-Maldonado et al. 2020). The 2 subunits of *pka* had multiple paralogs in both strains, with 4 copies of *bcy1* and 13 copies *tpk1*, many of these were upregulated in both strains. *byca* was upregulated in FRR5020. Another gene family associated with dimorphism is the 3 gamma units of G-protein coupled receptors: *gpb1* (mycelial growth), *gpb2* (yeast growth), and *gpb3* (mycelial growth). These were regulated differently in the 2 strains, with *gpb1* being downregulated and *gpb2* being upregulated in FRR5020, and *gpb2* being upregulated in VI04473. *crz1p* encodes a transcription factor that regulates a myriad of genes, including those involved in lipid metabolism (Cyert 2003). *crz1p* had putatively 7 copies in both strains, and 3 of these were upregulated. *crz1p* regulates a myriad of genes, including those involved in lipid metabolism (Cyert 2003). There are 4 copies of *pmc1* (vacuolar Ca2+ ATPase), which is involved in depleting the cytosol of Ca in the presence of elevated Ca levels. These were, as expected, downregulated under Ca starvation in both strains. Similarly, *pmr1* (plasma membrane ATPase related), which sequesters Ca into the Golgi apparatus, was downregulated in both strains under Ca starvation.

### No association between synteny and gene regulatory differences

The importance of gene order conservation in genome regulatory evolution has been the focus of much scientific debate (Kikuta et al. 2007; Poyatos and Hurst 2007; Bhutkar et al. 2008; von Grotthuss et al. 2010). We therefore investigated the role of genomic rearrangements on gene regulatory differences between FRR5020 and VI04473 using 3 different approaches. As above, we used both the Ca0 vs Ca1 and the P1 vs P4 contrasts to investigate gene expression differences between strains under more stressful vs less stressful conditions. First, we classified 1:1 orthologs between FRR5020 and VI04473 based on how many orthologous neighbors they share (0, 1 or 2 neighbors). If local microsynteny affects gene expression, we would expect a higher correlation of LFC between strains for genes with 2 neighbors, than for 1 or 0 neighbors. We found that for the genes with 0, 1, or 2, there was no differences in the correlation of LFC between strains, but it should be noted that the sample size between the 3 groups is quite skewed (0 neighbors: *n* = 160, 1 neighbor: *n* = 2,124, 2 neighbors: *n* = 6,875). Second, we classified each gene based on the size of the synteny block it is in, and the distance to its closest synteny breakpoint. We expected that the ambiguous or strain-specific DEGs would be in less conserved regions and thus smaller synteny blocks or closer to synteny breakpoints. However, we found no significant difference between the different DEG categories. Third, we investigated only genes very close to synteny breakpoints, hypothesizing that the regulatory landscape of these genes is more likely to be affected by rearrangements. We investigated the difference in LFC between strains as a function of distance to the closest synteny breakpoint in genomic regions <10,000 bp from a breakpoint. We observed a weak, negative relationship between the two, but this is not significant. In conclusion, we found little evidence for genomic rearrangements between strains being mechanistically linked to the low level of gene regulatory conservation between the 2 strains.

## Discussion

In line with 2 previous studies (Dzurendova et al. 2021a, 2021b) we have demonstrated that Ca starvation leads to divergent phenotypic effects in the 2 *Mucor circinelloides* strains, with VI04473 responding to this stressor with increased lipid accumulation and FRR5020 responding with increased carotenoid production (Fig. 1). We now also show that these strains have extremely divergent gene regulatory responses to Ca starvation (Fig. 3), and there are extensive genomic rearrangements between the 2 strains (Fig. 2).

In our study, we find very low conservation of Ca stress–induced gene expression responses, both genome-wide and for lipidome-genes (Fig. 3). The lack of gene expression conservation could be specific to the type of stress elicited by Ca starvation. Given that we observed a much higher gene expression correlation between strains when we compared 2 conditions that represent low stress (P1 and P4, Supplementary Fig. 7), this seems like a plausible explanation. This is concurrent with findings in yeast, where the conservation of gene expression under stress is dependent on the stressor, with heat shock responses being conserved whereas uracil limitation and rapamycin treatment had a higher number of genes with divergent expression (Guan et al. 2013). We found some notable differences between strains in the calcineurin signaling pathway under Ca starvation. This pathway is linked to the dimorphic switch between yeast and mycelial growth. We did not systematically quantify the level of yeast vs mycelial growth in this experiment, and it is possible that growth form differences are causing the strain differences in this pathway. The beta unit of calcineurin was downregulated in FRR5020, 1 of the alpha units (*cna_b*) and PKA subunits were upregulated in both strains. Upregulation of PKA can inhibit hyphal growth through inhibition of elongation factors under stress, such as anaerobiosis (Moriwaki-Takano et al. 2021; Homa et al. 2022). Taken together, the expression pattern of *gpb1*, *gpb2*, and *gpb3* suggests that FRR5020 had an increase in yeast growth and VI04473 in mycelial growth (Lee et al. 2013, 2015; Valle-Maldonado et al. 2020). This could explain the lack of conservation of genome-wide expression across strains. In the distantly related fungi *Candida albicans*, a remarkable transcriptomic plasticity enables the dimorphic switch and the yeast-to-hypha transition alone affects the expression of roughly 600 or 10% of all genes (Hnisz et al. 2012). Another potential contributor to the differences in gene expression we observed between strains is the transcription factor. In both strains, we found upregulation of multiple paralogs of the *crz1* transcription factor under Ca starvation, which could trigger a cascade of responses affecting many downstream genes (Lee et al. 2013). The triggering of the calcineurin pathway by Ca depletion seem to be conserved over large evolutionary distances as a similar response has been shown in yeast to ensure survival of the cells under stress (Bonilla et al. 2002). However, the direction of this response is not conserved and may be related to strain-specific triggers of dimorphic transition. This should be explored further in future gene regulatory investigations of *Mucor* species.

The overall conservation of expression of lipidome-genes is also not conserved between strains (Fig. 3b). When we examined these large differences in expression in different pathways leading to the production of carotenoids and lipids, we find several plausible mechanisms explaining the phenotypic divergence between strains. Carotenoid production involves relatively few genes (Fig. 4a), and we found that Ca starvation led to the down regulation of *crga,* a negative regulator of carotenogenesis in strain FRR5020, which could lead to higher production of carotenoids. In a study of *crga*-null mutants in *M. circinelloides*, the authors speculated that there are additional repressors of carotenoid synthesis in parallel to *crga* (Zhang et al. 2016). We discovered 2 paralogs to *crga* in both strains, which are possible candidates for buffering the effect of deleting *crga*. Curiously, we did not observe an upregulation of the structural carotenogenesis genes, *carb* and *carra*. Previous studies have documented that carotenoid production in *M. circinelloides* and *M. lusitanicus* is influenced by light (Naz et al. 2020), carbon source, temperature, salt concentration, and oxygen level (Nagy et al. 2014). Photoreceptors encoded by *wc* genes that are involved in light-induced carotenogenesis (Silva et al. 2006) were also differentially expressed in our experiment, even though we did not manipulate light. Thus, there is a possible link between calcium signaling and photoreception in *M. circinelloides*. To the best of the authors’ knowledge, this study presents the first evidence of Ca deficiency as a novel trigger for carotenoid production through regulations of the mevalonate pathway. Moreover, this is the first study to report the co-regulation of carotenoid and TAG synthesis under Ca deficiency, providing groundbreaking insights for strain development and optimization aimed at the simultaneous production of lipids and carotenoids.

Disentangling lipid accumulation in VI04473 under Ca starvation is more complicated as there are multiple, interconnected metabolic pathways involved in lipid production. These pathways compete for Acetyl-CoA utilization, an important metabolic intermediate (Sokołowska et al. 2023). In VI04473, the mevalonate and FAS pathways are downregulated under Ca starvation, and it is therefore plausible that lipid accumulation is mainly due to TAG synthesis. However, the regulatory pattern of genes in the TAG network in VI04473 does not fit with the model of TAG synthesis in yeasts like *S. cerevisiae*. We speculate whether TAG is formed directly from Acetyl-DHAP (Fig. 4c). This pathway may represent an alternate route for lipid synthesis, more closely resembling lipid metabolism seen in mammalian adipocytes than in yeasts (Hajra et al. 2000). Interestingly, similar mechanisms have been observed in algae, where Ca starvation also leads to increased lipid accumulation (Gorain et al. 2013). This indicates a potential common strategy across different groups of organisms for managing lipid storage under nutrient stress conditions, further highlighting the significance of Ca in regulating lipid metabolism. Whether this is homologous across these groups and lost in yeasts or the product of recurrent evolution remains to be tested. Furthermore, there are several lipidome-genes present in *S. cerevisiae* that are not present in *M. circinelloides* or Mucoromycotina as a whole, such as *ipt1*, *csh1*, *csg2*, *sur1*, *lpp1*, and *d5* (Supplementary Table 6) (Sokołowska et al. 2023). This also suggests that certain lipid pathways have been rewired in the *Mucor* lineage since it parted ways with its common ancestor with *S. cerevisiae.*

In this study, we detected multiple examples of paralogs with similar or different expression patterns (Figs. 3 and 4). Some of the differences in gene content between VI04473 and FRR5020 likely results from different retention of duplicates originating from an ancient whole-genome duplication in this lineage (Stajich 2016) as well as lineage-specific smaller scale gene duplication. Gene duplicates may have been maintained by selection to affect gene product dosage, and this could be the case for the *osi1* paralogs that are both significantly downregulated in VI04473 (Fig. 3d). Paralogs could have evolved different functions (subfunctionalization and/or neofunctionalization), such as the *faa2* and *ddl1* paralogs regulated in opposite directions in FRR5020 (Fig. 3c). These processes have likely contributed extensively to the phenotypic and gene regulatory divergence between these 2 lineages, especially related to lipid and carotenoid production. The turnover of lipidome-genes and their role in adaptation to different lifestyles is well documented in *Mucoromycota* (Lebreton et al. 2020; Sokołowska et al. 2023) and fungi, in general (Corrochano et al. 2016; Naranjo-Ortiz and Gabaldón 2020; Wu and Cox 2021). In this study, we have uncovered several candidate gene duplicates that could be related to such a lifestyle switch and would be interesting subjects of future functional studies.

In addition to differences in gene content between these 2 *M. circinelloides* strains, we also observed extensive genomic rearrangements between strains (Fig. 2i). We found that these synteny disruptions seem to have had no effect on the gene regulatory differences between strains, at least not related to Ca or P levels. Similar observations have been made in yeasts, where the co-expression of genes is a poor predictor of conservation of microsynteny or local gene neighborhoods (Poyatos and Hurst 2007). However, to get a better overview of the effect of genome rearrangements on gene regulation, we would need to investigate gene expression differences over broader environmental conditions as well as include more species to reflect different evolutionary distances (Martin and Fraser 2018). Extensive changes to genome organization have been shown to facilitate adaptations to new niches in other fungi (Naranjo-Ortiz and Gabaldón 2020). Large-scale synteny comparisons have been carried out in other fungal lineages (Hane et al. 2011; Li et al. 2022), but never in *Mucoromycota*. Between FRR5020 and VI04473, most gene pairs have remained neighbors, and we would therefore most likely not detect purifying selection to keep them linked to conserve co-expression (Poyatos and Hurst 2007). Furthermore, the extensive rearrangements we observe in *M. circinelloides* (Fig. 3) could contribute to reproductive isolation and speciation in this genus, thus indirectly influencing divergence in evolutionary rewiring of gene regulatory networks. The extensive genomic rearrangements between VI04473 and FRR5020 is concurrent with comparisons between genomes of a high lipid-producing strain, WJ11, and a low lipid-producing strain, CBS 277.49, which also display large phenotypic differences (Tang et al. 2015). We propose that it is possible that these strains may in fact represent different species. Delineating species in fungi is challenging, and there is currently no consensus as to what species concept or criteria should be used (Xu 2020; Boekhout et al. 2021; Chethana et al. 2021). These criteria—from morphological, ecological, and biological to phylogenetic—often represent at what stage 2 lineages are in a continuous process of divergence. Investigating whether these 2 *M. circinelloides* strains mate and recombine, ie are biological species or not, equivalent to what has been done for other strains (Wagner et al. 2020), would shed light on whether the rearrangements we observe (Fig. 3) still segregate within *M. circinelloides* populations. Another way would be to investigate *M. circinelloides* strains in a population genomics framework. Evaluating the taxonomic status of different *Mucor* strains in a systematic way using multiple species recognition criteria is beyond the scope of the current study. Regardless of where these 2 strains are on the speciation spectrum, this investigation still gives fascinating insight into genomic, gene regulatory, and phenotypic/ecological divergence between recently diverged lineages of *M. circinelloides*.

### Future perspectives

Our study highlights the value of genome-wide investigations of metabolic pathways, both to gain and deeper understanding on the evolution of the diverse *M. circinelloides* strains and to optimize production of valuable metabolites for industrial purposes. The value of such genomic studies has been well documented in the model species yeast *S. cerevisiae* (Hittinger et al. 2015; Riley et al. 2016). For most species of *Mucoromycota*, we still have very little knowledge about their genome biology, despite their ubiquitousness in nature, ecological significance, medical relevance, and biotech potential. A major challenge now is to link genomic variation to organismal phenotypes through functional genomic studies in species that remain underutilized by industry and science. Such studies can ultimately lead to a detailed understanding of gene networks that is needed for optimization and engineering of bio-based production.

## Supplementary Material

jkaf207_Supplementary_Data

## Data Availability

Genome assemblies and annotation generated for this paper are on ENA (VI04473: PRJEB96070; and FRR5020: PRJEB96011). RNA-seq data are deposited in the ArrayExpress database, accessions E-MTAB-13309, E-MTAB-15537, E-MTAB-15566 and E-MTAB-15567. Supplementary File 1 contains all supporting tables and their descriptions. Data and custom scripts are available at https://doi.org/10.25387/g3.29948891. Supplemental material available at G3 online.
