# Supplementary material for: Calcium starvation leads to strain-specific gene regulation of lipid and carotenoid production in Mucor circinelloides
Source: G3 (Bethesda). 2025 Sep 6;15(11):jkaf207. doi: 10.1093/g3journal/jkaf207 (PMC12611239; doi:10.1093/g3journal/jkaf207)
Supplement: jkaf207_Supplementary_Data [file jkaf207_supplementary_data.zip › Supplementary_Figures_G3-2025-406071.docx]

Supporting Information: Calcium starvation leads to strain-specific gene regulation of lipid and carotenoid production in *Mucor Circinelloides*

Baalsrud HT^1*^, Byrtusova D^1^, To TH^1^, Larsen IE^1^, Bøe VA^1^, Grønvold L^1^, Fu J^1^, Árnyasi M^1^, Shapaval V^1^, Sandve SR^1^

Affiliation and address:
1. Norwegian University of Life Sciences, 1433 Ås, Norway

*Corresponding author: [helle.tessand.baalsrud@nmbu.no](mailto:helle.tessand.baalsrud@nmbu.no)


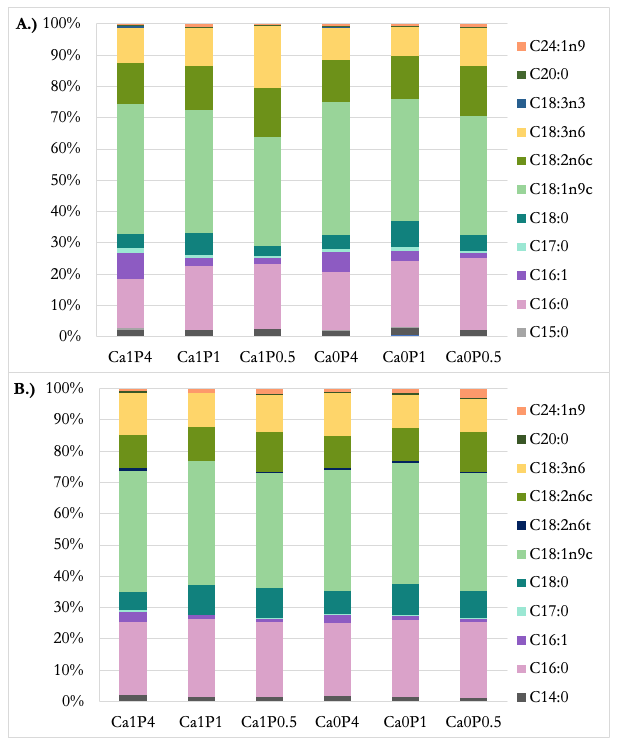


**Supplementary Figure 1**: Fatty acids profiles for A.) VI04473 and B.) FRR5020 at the six treatments with different combinations of Ca and P levels. Each fatty acid is colored according to legend.


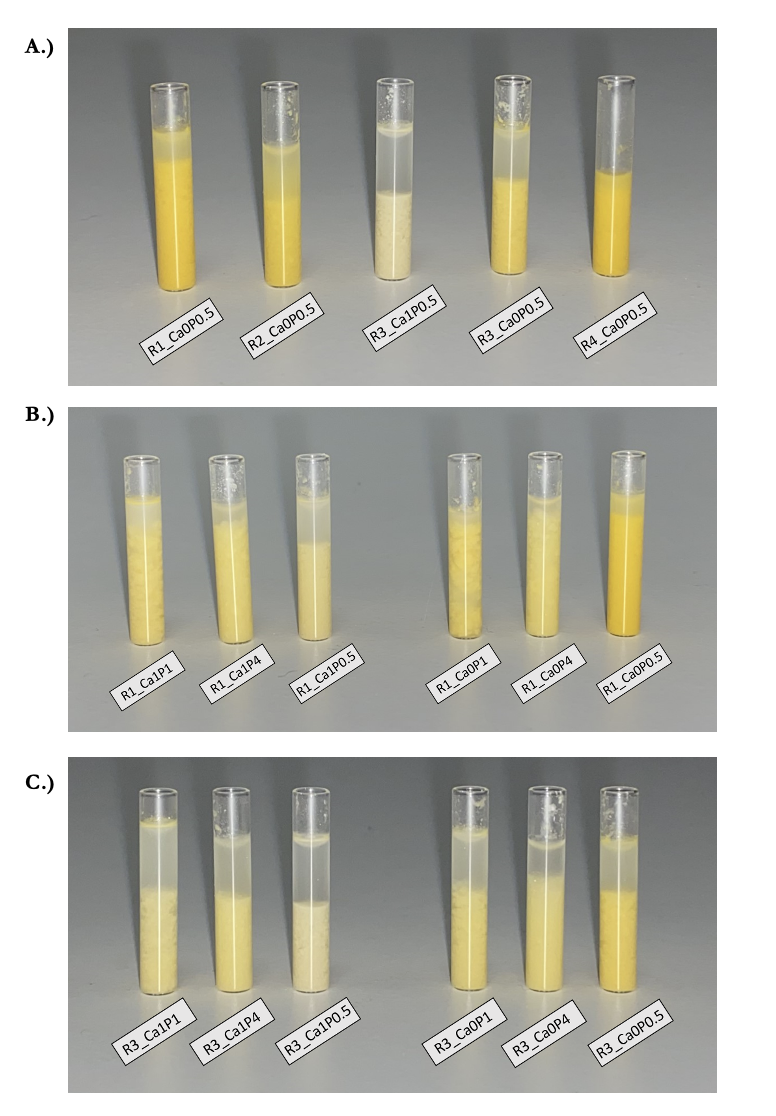


**Supplementary Figure 2:** Pictures of *Mucor circinelloides* strain FRR5020 tissue samples at different combinations of Ca and P treatments. R denotes replicate.


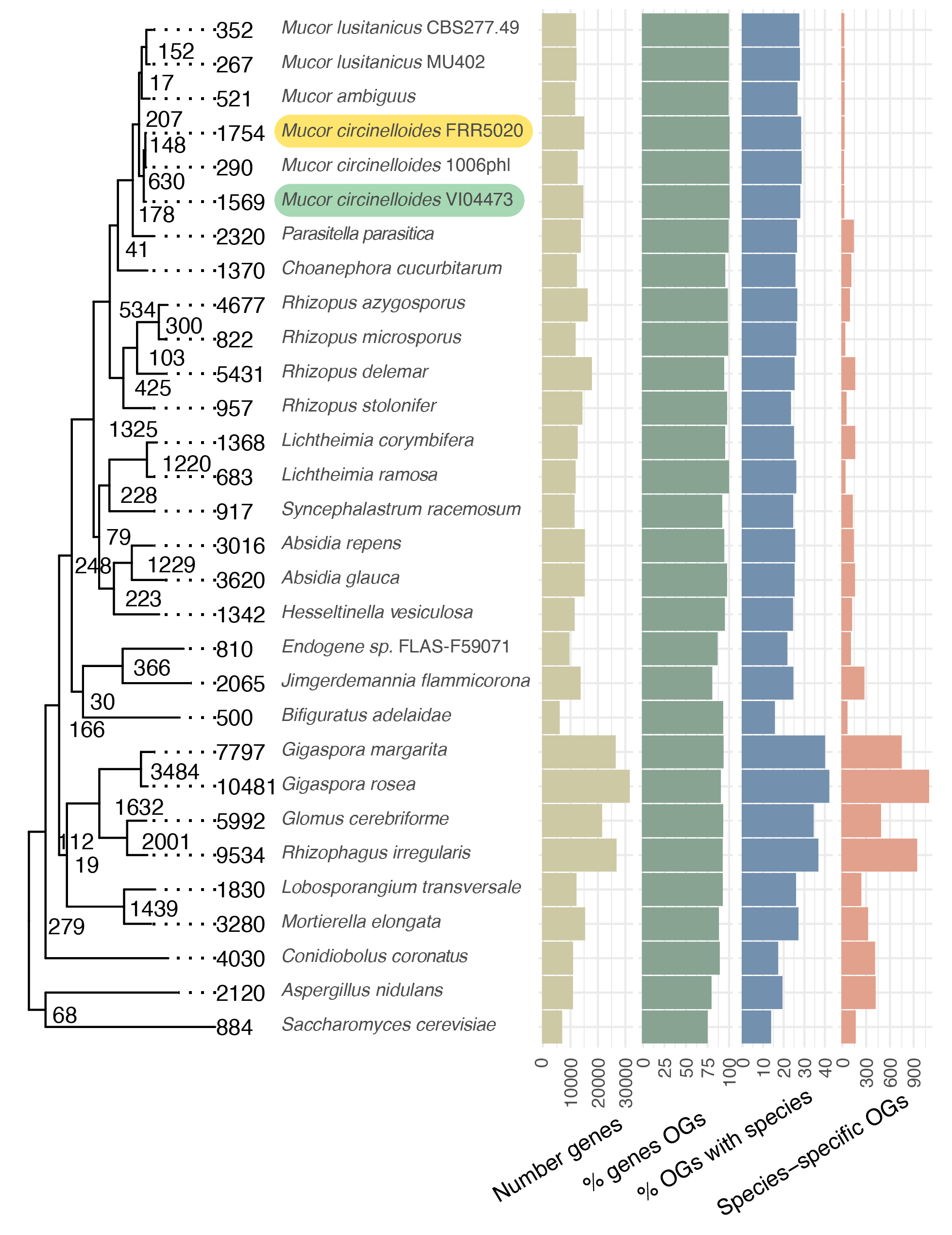


**Supplementary Figure 3: Phylogenetic tree and Orthofinder results for 27 strains/species of fungi in the Mucoromycota division.** *Conidiobolus coronatus* (division: Entomophthoromycota), *Aspergillus nidulans* and *Saccharomyces cerevisiae* (division: Ascomycota) are outgroup species. The main strains investigated in this study highlighted in yellow (FRR5020) and turqoise (VI04473). The strain MU402 is derived from the strain CBS277.49 [(Corrochano *et al.* 2016)](https://sciwheel.com/work/citation?ids=2546367&pre=&suf=&sa=0&dbf=0). Phylogenetic tree was generated by Orthofinder. The number of gene duplications is denoted at each tip and node. Bar plots show the number of genes placed in orthogroups (OGs), the percentage of genes in orthogroups, the percentage of orthogroups containing that species, and how many species-specific orthogroups there are.


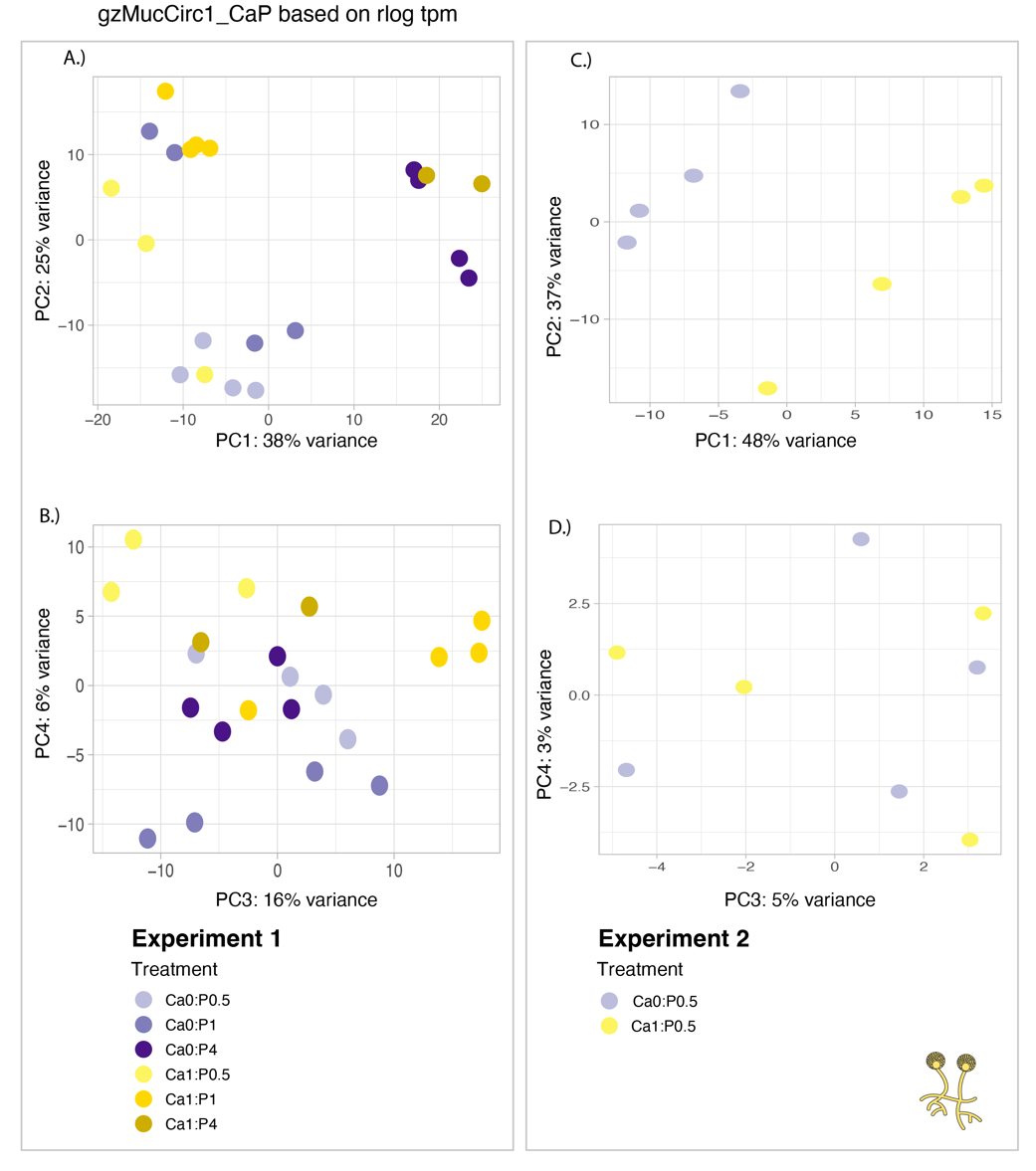


**Supplementary Figure 4:** PCA plots for FRR5020 from experiment 1 (Ca and P) and experiment 2 (only Ca). Samples are colored according to legend.


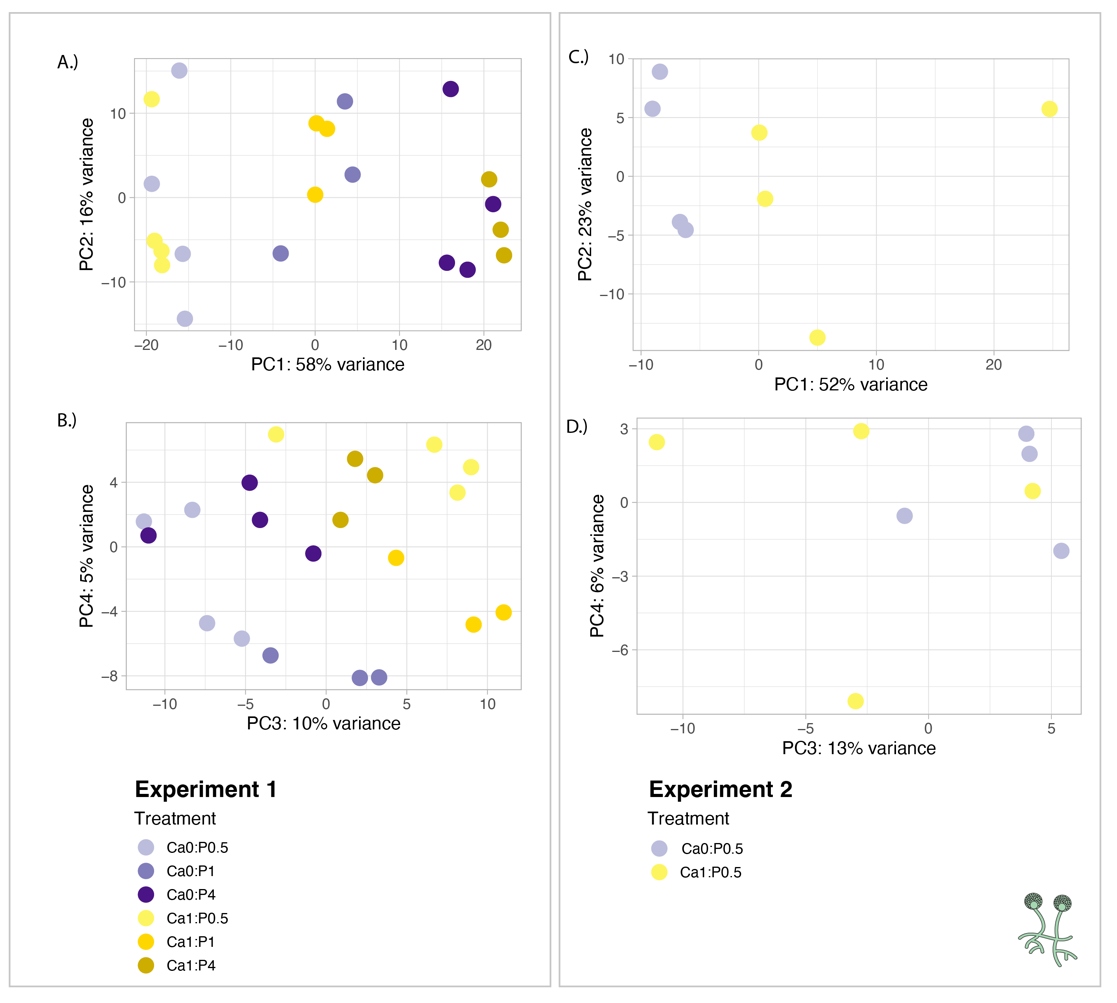


**Supplementary Figure 5:** PCA plots for VI04473 from experiment 1 (Ca and P) and experiment 2 (only Ca). Samples are colored according to legend.


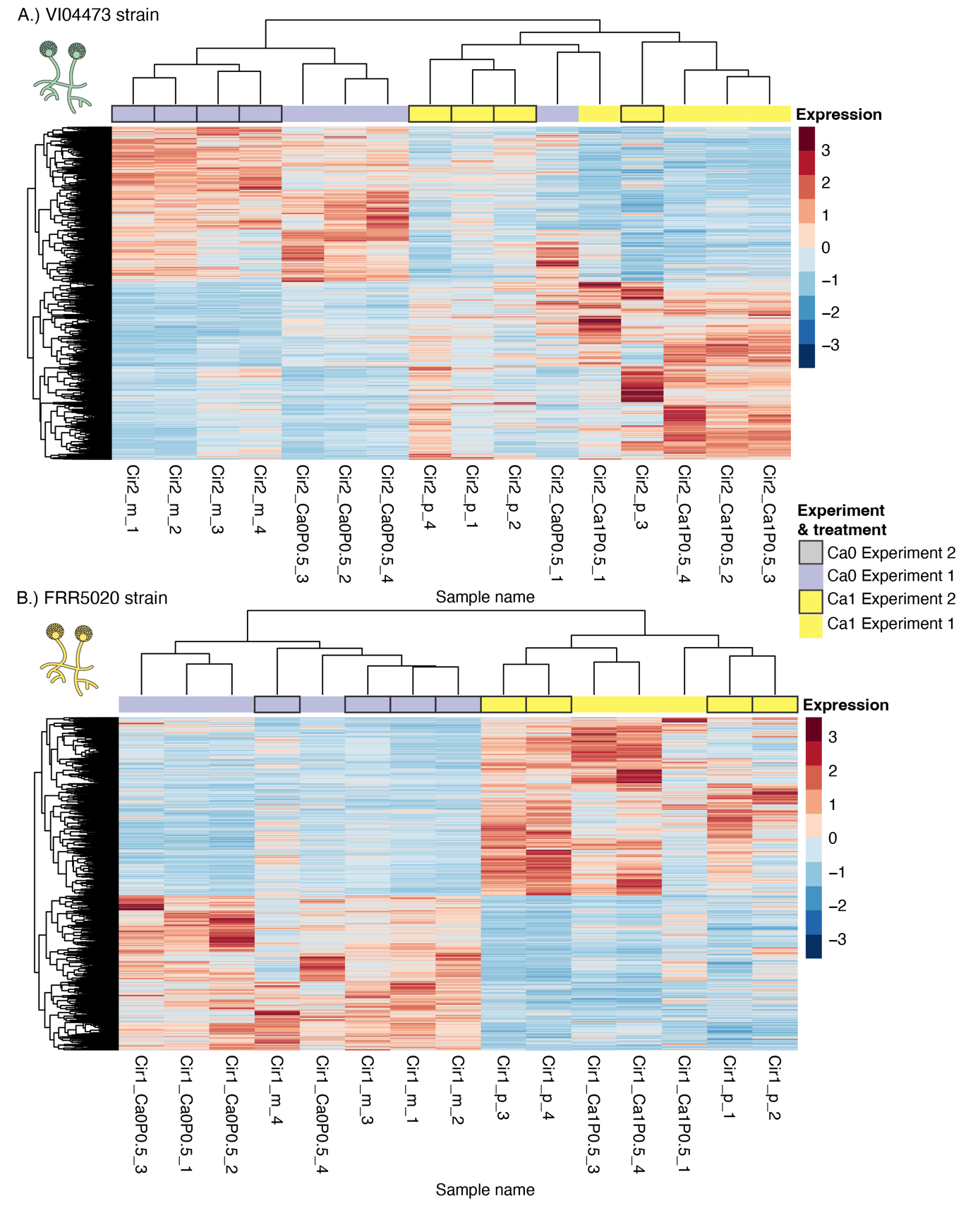


**Supplementary Figure 6:** Heatmaps of gene expression of significant DEGs (padj < 0.1) in *Mucor circinelloides* strains A.) VI04473 and B.) FRR5020. Differential expression (log2FoldChange) is colored according to legend. The dendrogram at the top shows a hierarchical clustering of the samples. Samples are from two separate experiments; Experiment 1 and Experiment 2, with two treatments; Ca0 or Ca1, according to legend. All samples have P0.05. Sample names for the different technical replicates are shown at the bottom.


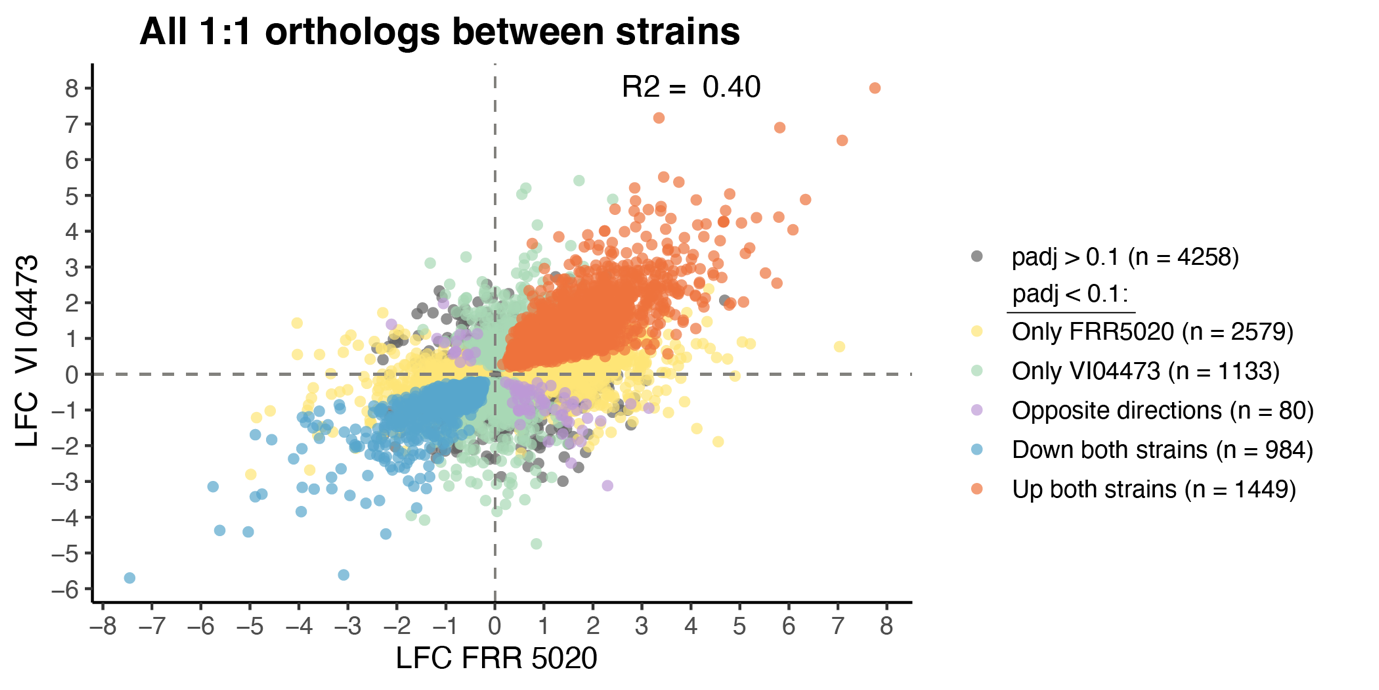


Supplementary Figure 7: Correlation of LFC between VI04473 and FRR5020 for all 1:1 orthologs in Genes are grouped according to legend. Differential expression analyses was carried out for the contrast between normal ang high phosphate (P1 vs P4).


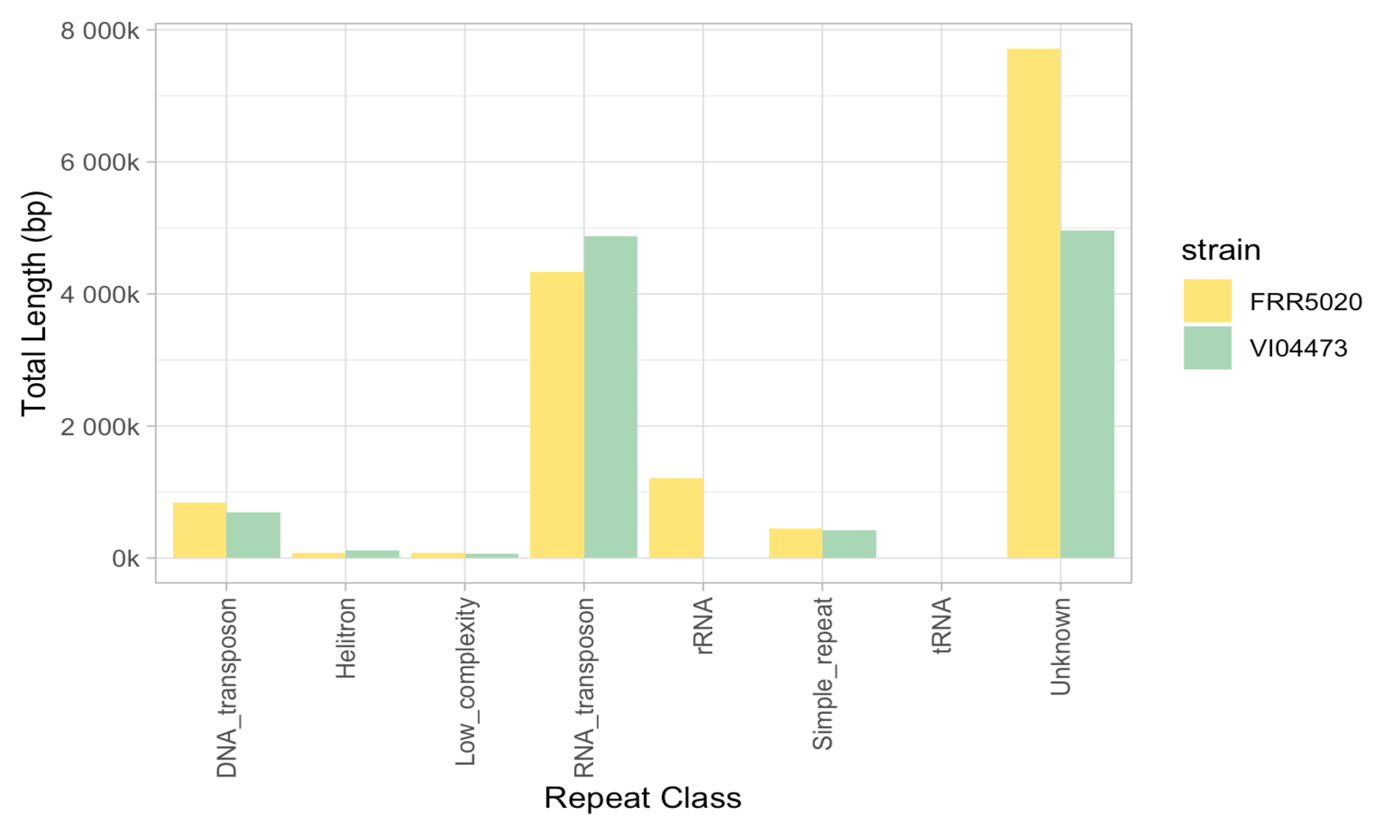


**Supplementary Figure 8**: Repeat content in the two Mucor circinelloides strains. The y-axis shows the summed length of all repeat elements within a repeat class (in kilo base pairs). The colors of the bars represent the two strains according to the legend.
